# A Case of Pericostal Tuberculosis Discovered Incidentally on an Abdominal MRI

**DOI:** 10.1002/rcr2.70212

**Published:** 2025-05-20

**Authors:** Kei Kanata, Toshihiro Shirai, Shoma Matsushita, Yutaro Ito, Koshiro Ichijo, Masahiro Uehara

**Affiliations:** ^1^ Department of Respiratory Medicine Shimada General Medical Center Shizuoka Japan; ^2^ Department of Respiratory Medicine Shizuoka General Hospital Shizuoka Japan

**Keywords:** magnetic resonance imaging, thoracoscopic resection, tuberculosis

## Abstract

A mobile pleural mass on an MRI was incidentally detected in a 45‐year‐old woman. Thoracoscopic resection revealed caseous necrosis and PCR‐confirmed *Mycobacterium tuberculosis* , leading to a diagnosis of pericostal tuberculosis. This rare case emphasises considering pericostal tuberculosis in the differential diagnosis of pleural tumours if there are suspicious MRI features.

## Graphical abstract

A mobile pleural mass on an MRI was incidentally detected in a 45‐year‐old woman. Thoracoscopic resection revealed pericostal tuberculosis with PCR‐positive *Mycobacterium tuberculosis* . This rare case emphasizes considering pericostal tuberculosis in the differential diagnosis of pleural tumors if there are suspicious MRI features.

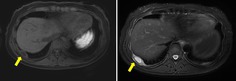

A 45‐year‐old woman undergoing a medical examination was suspected of a pancreatic tumour by ultrasound. She underwent an MRI of the abdomen, which revealed no abnormality in the pancreas but a 3‐cm pericostal tumour located in the pleural site at the level of the 11th rib (Figure 1). T1‐weighted MRI revealed low intensity, and T2‐weighted MRI revealed high intensity. An enhanced CT scan on a later day revealed the tumour had moved to the level of the ninth rib. The internal site of the tumour was not enhanced, and the peripheral site of the tumour was enhanced (Figure 2). The patients underwent video‐assisted thoracoscopy, revealing a white fluid leaked from the tumour (Figure 3). Histopathology of the tumour demonstrated a mass containing caseous necrosis and Langhans giant cells (Figure 4), and the tuberculosis PCR was positive. Thus, we diagnosed pericostal tuberculosis.

**FIGURE 1:**
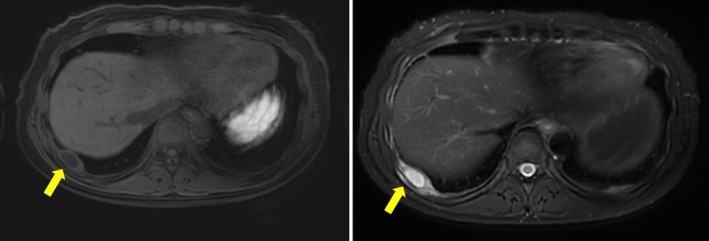
Magnetic resonance imaging (MRI) on presentation, demonstrating an almost 3‐cm tumour at the level of the 11th rib, which was low intensity by T1 and high intensity by T2. The tumour was marked with an arrow.

**FIGURE 2:**
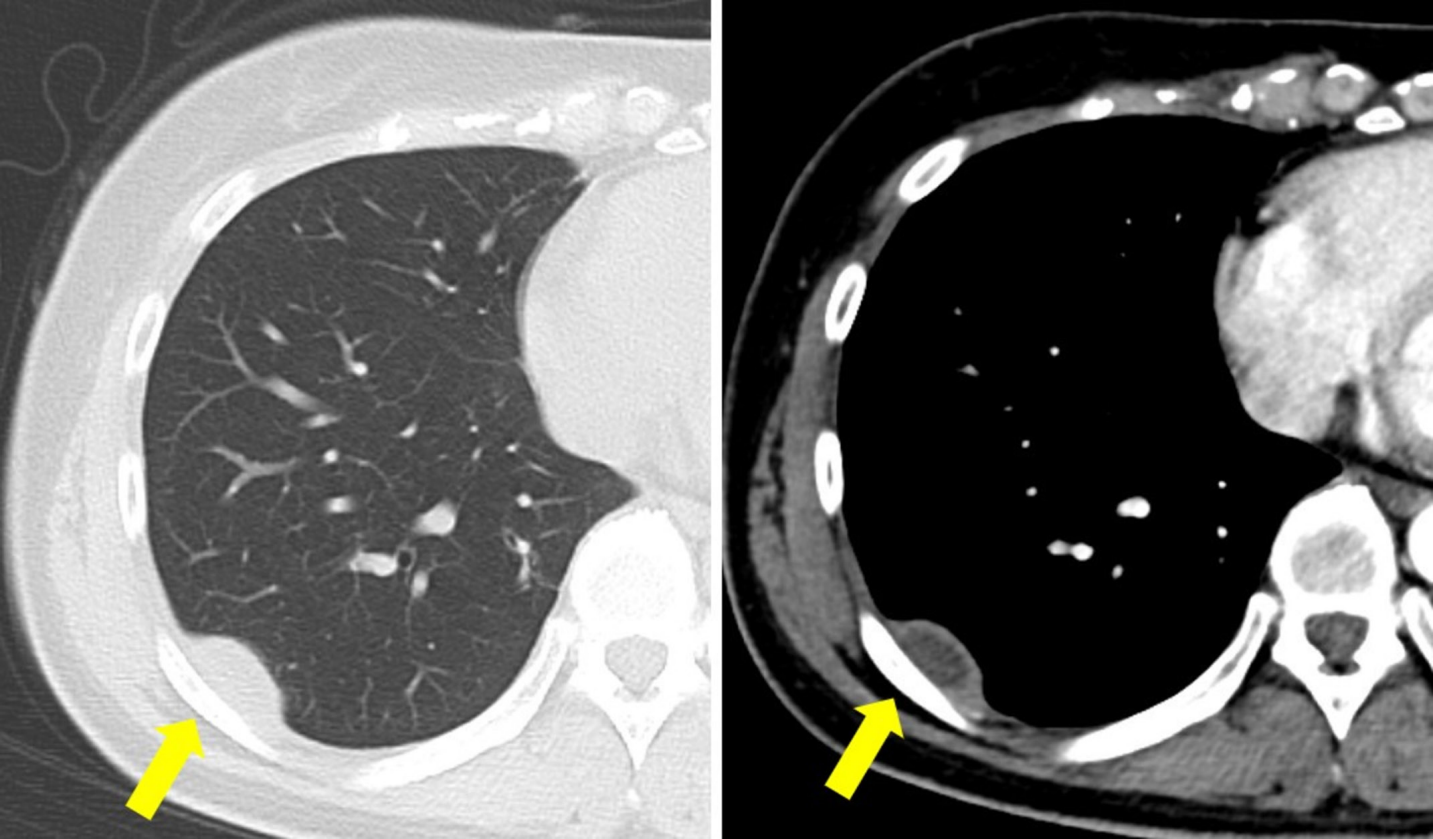
Enhanced computed tomography (CT) scan, demonstrating a tumour moving to the level of number 9th rib. The internal site of the tumour was not enhanced, and the peripheral site of the tumour was enhanced. The tumour was marked with an arrow.

**FIGURE 3:**
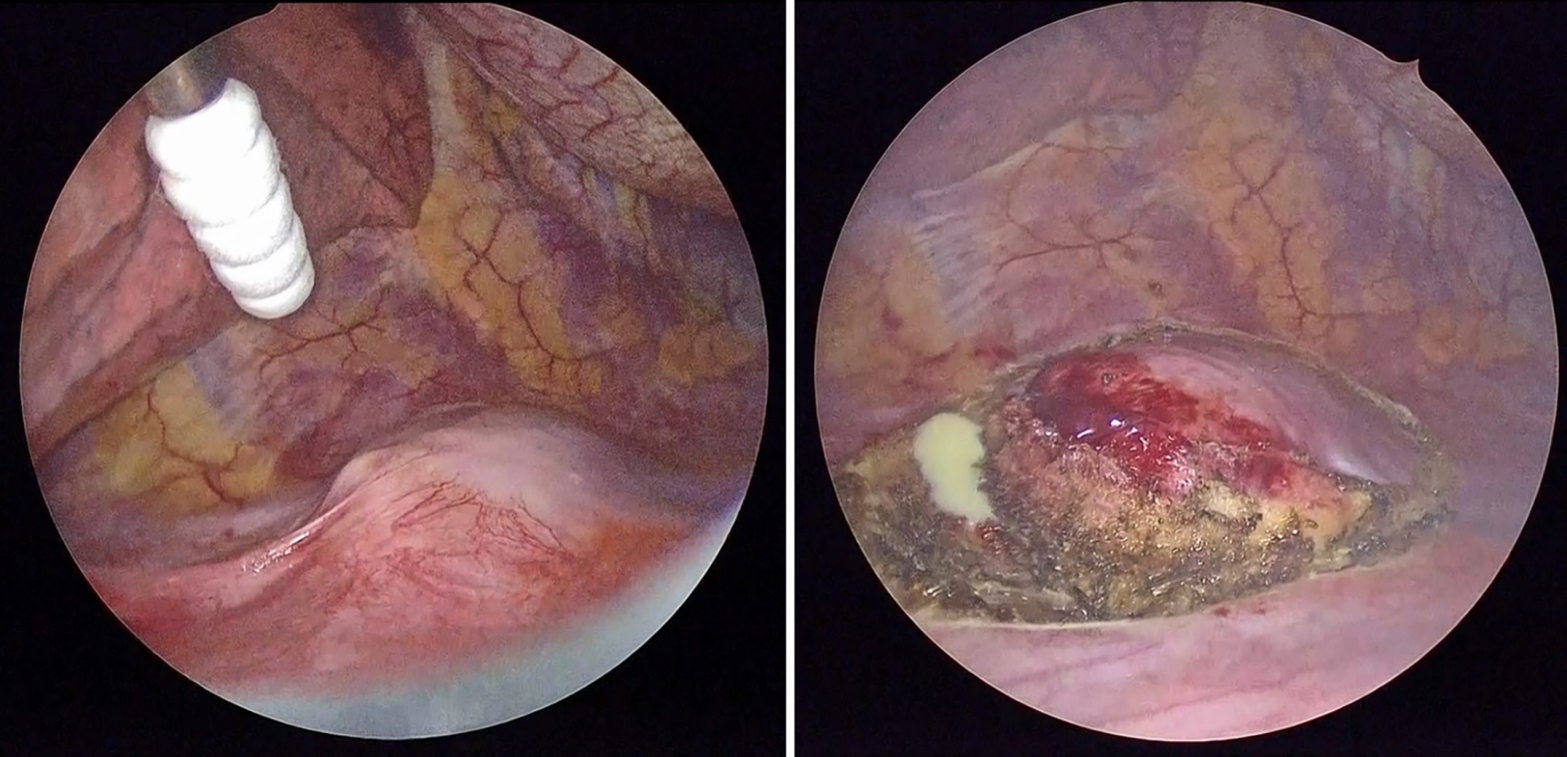
The right side thoracoscope, demonstrating the chest wall tumour; a white fluid leaked from the tumour.

**FIGURE 4:**
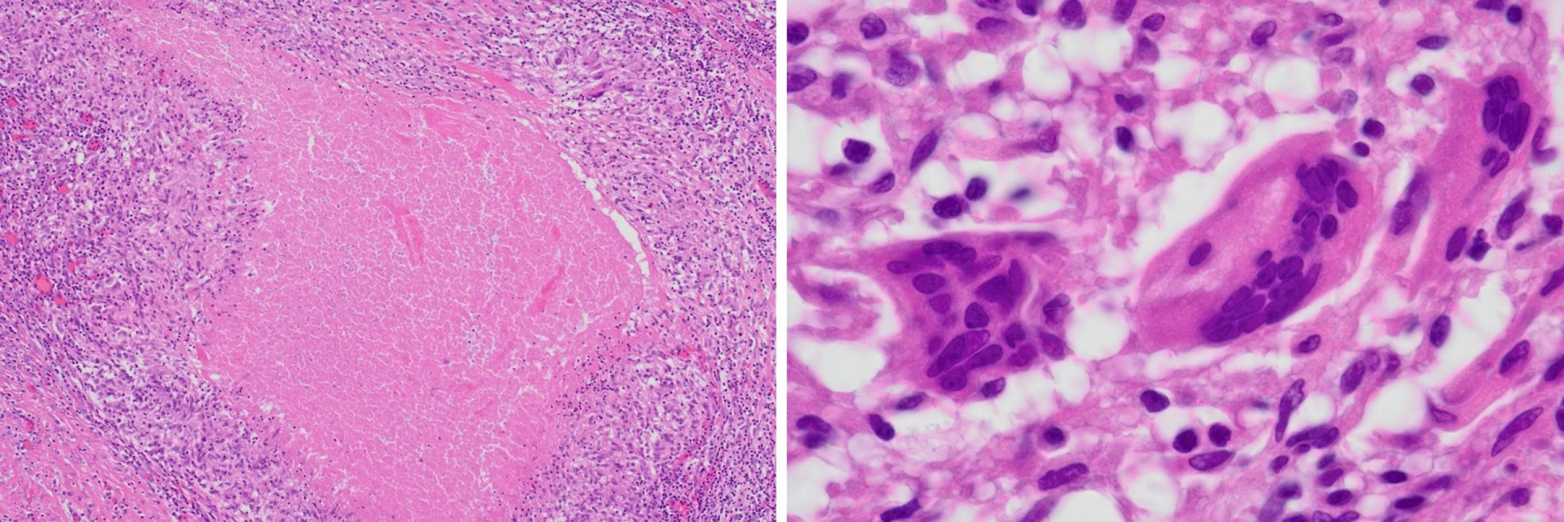
The histopathology of the tumour, demonstrating a mass containing caseous necrosis and Langhans giant cells. (HE staining).

Pericostal tuberculosis is rare, likely resulting from intercostal lymph node TB extension [1]. It may migrate and attach to the pleura. These imaging findings suggest including periosteal tuberculosis in the differential diagnosis [2]. What was interesting in this case was that the findings were discovered incidentally on an abdominal MRI performed to examine for abdominal disease.

## Author Contributions

All authors are legitimate authors on the manuscript; all authors have read and approved the final version of the submitted manuscript.

## Consent

The authors declare that written informed consent was obtained for the publication of this manuscript and accompanying images and attest that the form used to obtain consent from the patient complies with the Journal requirements.

## Conflicts of Interest

The authors declare no conflicts of interest.

## Data Availability

Data sharing not applicable to this article as no datasets were generated or analysed during the current study.
